# TOO HOT OR TOO COLD? EXPOSURE TO EXTREME TEMPERATURES AND COGNITIVE FUNCTION IN OLDER ADULTS

**DOI:** 10.1093/geroni/igz038.104

**Published:** 2019-11-08

**Authors:** Anam M Khan, Philippa Clarke, Jessica Finlay, Carina Gronlund, Robert Melendez, Ketlyne Sol, Suzanne Judd, Virginia Wadley

**Affiliations:** 1 University of Michigan, Ann Arbor, Michigan, United States; 2 Department of Biostatistics, University of Alabama at Birmingham, Birmingham, Alabama, United States; 3 Department of Medicine, University of Alabama at Birmingham, Birmingham, Alabama, United States

## Abstract

Research on temperature and cognition is sparse, including effects of outdoor air temperature on cognitive testing performance. Furthermore, little is known about the modifying role of region and seasonality in temperature-cognition associations. We linked daily temperature data from National Oceanic and Atmospheric Administration weather stations to REGARDS participants by cognitive assessment date. Controlling for season, generalized linear models including spline terms for temperature showed an adverse effect of hotter temperatures on cognition. At higher temperatures (30°C vs 0°C), there was a significant decrease in cognitive performance on the Word List Learning test (β=-0.68; 95% CI: -1.1, -0.25). Results also show regional differences in testing scores on hotter and colder days. The findings provide new understanding of cognitive susceptibility to extreme temperatures and factors that exacerbate or buffer this association. This can inform development of evidence-based public health guidelines and mitigation strategies aimed at reducing temperature-related morbidity in older adults.

